# Husserlian phenomenology in Korean nursing research: analysis, problems, and suggestions

**DOI:** 10.3352/jeehp.2020.17.13

**Published:** 2020-04-21

**Authors:** Hye-Kyung Kim, Myunghee Jun, Stephanie Rhee, Michael Wreen

**Affiliations:** 1 Department of Philosophy, University of Wisconsin-Green Bay, Green Bay, WI, USA; 2 Department of Nursing and Health Studies, University of Wisconsin-Green Bay, Green Bay, WI, USA; 3 Social Work Professional Programs, University of Wisconsin-Green Bay, Green Bay, WI, USA; 4 Department of Philosophy, Marquette University, Milwaukee, WI, USA

**Keywords:** Korea, Nursing research, Methodological studies, Qualitative research, Philosophy

## Abstract

**Purpose:**

This paper is a critical review of the descriptive phenomenological methodology in Korean nursing research. We propose constructive suggestions for the improvement of descriptive phenomenological methodology in light of Husserl’s phenomenological approaches.

**Methods:**

Using the keywords of ‘phenomenology,’ ‘experience,’ and ‘nursing,’ we identify and analyze 64 Korean empirical phenomenological studies (selected from 282 studies) published in 14 Korean nursing journals from 2005 to 2018. The PubMed and the Korea Citation Index were used to identify the studies.

**Results:**

Our analysis shows that all the reviewed articles used Giorgi’s or Colaizzi’s scientific phenomenological methodology, without critical attention to Husserl’s philosophical phenomenological principles.

**Conclusion:**

The use of scientific phenomenology in nursing research, which originated in North America, has become a global phenomenon, and Korean phenomenological nursing research has faithfully followed this scholarly trend. This paper argues that greater integration of Husserlian phenomenological principles into scientific phenomenological methodology in nursing research, such as participant-centered bracketing and eidetic reduction, is needed to ensure that scientific phenomenology lives up to its promise as a research methodology.

## Introduction

### Background/rationale

As philosophy and research methodology, phenomenology has laid the foundation for theoretical knowledge and methodological clarity and rigor in qualitative nursing research [1,2]. Nursing researchers have adopted phenomenological approaches for their qualitative research framework to better understand human phenomena in the context of nursing practice. Such research requires both humanistic philosophy and scientific precision. However, it has been challenging for nursing researchers to apply complex phenomenological principles and concepts to empirical nursing research [2]. In an effort to clarify philosophical and empirical complexities of phenomenology in nursing research, a descriptive phenomenological research methodology, which we call scientific phenomenology in this article, has been a global scholarly trend. Korean phenomenological nursing research has faithfully followed this trend.

In this article, ‘scientific phenomenology’ refers to the descriptive phenomenological methodology of Colaizzi [3] in 1978 or Giorgi [4] in 1997. Both use Husserlian philosophical phenomenology as its epistemic foundation. Scientific phenomenology in nursing research aims at discovering and describing the essential meanings of people’s lived experiences [5]. It includes researcher’s bracketing and participants’ interviews as data collection and employs a stepwise data analysis. In the late 1990s, the use of scientific phenomenology as scientific research methodology was criticized for disregarding fundamental principles of Husserlian philosophical phenomenology and misinterpreting its key concepts by Crotty [6] in 1996 and Paley [7] in 1997. In their systematic review of phenomenological nursing research published 10 years later, Norlyk and Harder [2] found that scientific phenomenology has been the major research methodology of descriptive phenomenological research in nursing, conceptually separated from its philosophical underpinnings in Husserlian phenomenology. There are 3 major principles of Husserl’s phenomenology in relation to scientific phenomenology: phenomena, bracketing, and eidetic reduction. For Husserl [8], ‘phenomena’ refer to objects as they appear in consciousness. As Husserl [8] in 1965 says, objects become phenomena as they are “perceived, remembered, expected, represented pictorially, imagined, identified, distinguished, believed, opined, evaluated, and etc.” ‘Knowledge’ for Husserl [8] is obtained by apprehending the essences of the conscious experience by the person who experiences the conscious acts. Bracketing involves suspending judgment about the natural world and its existence. Husserl [8] believes that the analysis of conscious experience must be conducted from a first-person point of view. From a first-person perspective, one cannot be sure that the object one perceives or experiences (e.g., a table) exists apart from experience itself. For that reason, Husserl [8] claims that assumptions about the existence of objects of experience outside the experience (e.g., physical objects) must be suspended.

Eidetic reduction involves the identification and removal of any contingent and accidental features of our experiences to intuit the invariant and necessary features of experiences [9]. The intuition of essential features or essences of our experience proceeds through what Husserl [10] in 1977 calls free variation in imagination. The essential features of an object in conscious experience are the invariant, necessary, and universal features which the object is inconceivable. Thus eidetic reduction leads to first-person intuitions of essences of conscious experience, essences which cannot be changed and without which the experience would not be the experience it is. In Husserlian phenomenology, the essential meaning of the phenomenon is achieved through first-person bracketing and first-person eidetic reduction by the person who experiences the phenomenon. By way of contrast, scientific phenomenology employs third-person (researcher-centered) bracketing: researchers freeing themselves from their own theoretical presuppositions and biases in data collection. In addition, researchers employ third-person stepwise categorical reduction of the data collected from participants’ lived experience.

Scientific phenomenology has been the major methodology in Korean descriptive phenomenological nursing research. The use of scientific phenomenology originated in the late 1980s. The method quickly took hold and gained ascendancy as a preferred methodology. Gong [11] in 2004 and Lee [12] in 2005 are the first Korean scholars to cast a critical eye on the merits of scientific phenomenology. Lee [12] claimed that scientific phenomenology is a promising nursing research methodology based on Husserlian phenomenological principles. Lee [12] observed, nevertheless, the need for further development in the methodology. Despite critical remarks of Lee [12] on scientific methodology, however, little attention has been paid to developing the methodology of scientific phenomenology in Korean nursing research, and virtually no progress has been made. The need for further development of scientific phenomenology was also expressed by Giorgi [13] in 2000. Giorgi [13] asked descriptive phenomenological researchers “not to stay away from Edmund Husserl or other phenomenologists.”

### Objectives

In this article, we critically review the major features of current descriptive phenomenological nursing studies published from 2005 to 2018 in Korea. We also propose some suggestions to strengthen the epistemic foundation of scientific phenomenological nursing research in Korea.

## Methods

### Ethics statement

It is the literature-based study; therefore, no approval from the institutional review board is required.

### Research design

This study is a critical literature review of descriptive phenomenological nursing research in Korea.

### Search procedure

**Fig. 1.**
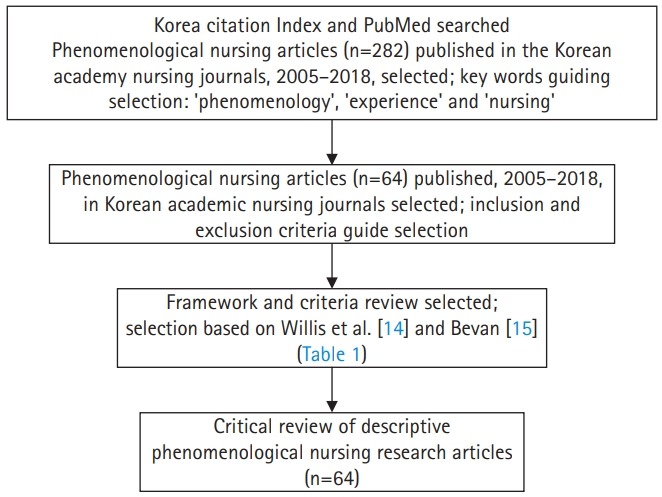
Flowchart of this critical review process.

We investigated Korean phenomenological nursing researches by analyzing 64 descriptive phenomenological research articles. The articles were identified by consulting the PubMed and the Korea Citation Index (Fig. 1). The articles were published from 2005 to 2018 in 14 Korean journals. The search was last conducted on December 31, 2018.

### Inclusion and exclusion criteria

Two hundred and eighty-two articles were identified by the keywords ‘phenomenology,’ ‘experience,’ and ‘nursing.’ The year 2005 was selected as the initial year for the search. That year was chosen, based on first critical reflections of Gong [11] and Lee [12] on the methodology of descriptive phenomenological nursing research. Their researches were geared to identify the merits, limitations, and possible problems with scientific phenomenology as a research methodology. Their critiques were the beginning of critical phenomenological nursing research in Korea. Research articles which employed interpretive or hermeneutic phenomenology were excluded from our study, because our purview is confined to descriptive phenomenological nursing research in Korea based exclusively on Husserl’s phenomenology.

### Data extraction and analysis

Four researchers read the abstracts of 282 articles written in English. Sixty-four were selected for analysis and evaluation (Fig. 1). The full texts of all 64 articles were then read by 3 Korean-American researchers. A framework for analysis was then established (Table 1). The framework included general characteristics, features of descriptive qualitative research, and phenomenological research features. The general characteristics of the articles were identified in terms of their year of publication, type of participants, and the method of sampling. The features of descriptive qualitative research in the articles were identified in terms of interview type, interview question type, method of enhancing the quality of the interview, and validity criteria. The features of phenomenological research were identified in terms of the purpose of the research, bracketing, method of data analysis, and eidetic reduction for essential meaning. Researchers’ suspension of pre-assumptions and presuppositions without an explicit mention of ‘bracketing’ was taken to include implicit bracketing.

The framework and criteria/features of the critical review recorded on Tables 1 and 2 were based on those found in ‘Distinguishing features and similarities between descriptive phenomenological and qualitative description research’ of Willis et al. [14] and ‘A method of phenomenological interviewing’ of Bevan [15].

Sixty-four articles were analyzed by 4 researchers in accordance with the criteria of the framework (Table 1). The number of articles exhibiting each feature in the framework was identified (Table 2). There was 96.8% agreement among researchers in their assessments. The 3.2% disagreement was resolved through discussions among the 4 researchers. Discussions yielded unanimity and thus established 100% inter-rater reliability on assessment.

## Results

### Characteristics of the articles

Phenomenological nursing research has been actively and continuously pursued in Korea since 2005. The most active year was 2017, with 11 articles (17.2%) appearing. There were several types of participants, with patients and nurses constituting the largest group (65.6%). Sampling was conducted in diverse ways. Purposive sampling was most popular, while 25 articles (39.1%) do not mention their methods of sampling.

### Features of descriptive qualitative research in the articles

The articles exhibited the general features of descriptive qualitative research. The first feature was the interview type. All surveyed articles (100%) used in-depth individual interviews as their source of data. Questions asked during the interviews were of various types. Forty articles (62.5%) used open and/or semi-structured questions, while 23 articles (35.9%) did not mention question type. Thirty-eight articles (59.4%) mentioned that efforts were made to provide a comfortable environment to the participants in order to enhance the quality of the interview data. Thirty-seven articles (58.8%) mentioned researchers’ efforts to establish a rapport with participants. In 20 articles (32.3%), researcher’s non-interference in participants’ narration of their experiences was cited as a way to enhance the quality of research data. For validity assurance, either the criteria of Lincoln and Guba [16] or the criteria of Sandelowski [17] were used in 46 articles (71.9%), while for truthfulness validation on results of research, either member check or peer check was used by researchers in 48 articles (75.0 %).

### Features of scientific phenomenological research in the articles

Fifty of 64 articles (78.1%) identified the purpose of the research as an in-depth understanding of the essential meaning and structure of the experience of participants. The remaining 14 articles (21.9%) identified a rich description or an exploration of the experience of participants as the aim of the research. Methods of data analysis are explicitly identified in all 64 articles. Forty-seven articles (73.4%) exclusively followed the method of Colaizzi [3], and 17 articles (26.6%) exclusively follow the method of Giorgi [18].

Fifty-three articles (82.8%) either explicitly or implicitly included bracketing in their protocol. Bracketing was used in data collection and/or data analysis. In 44 articles (68.8%) bracketing was used at the stage of data collection (interview). In another 35 articles (54.7%) bracketing was used at the stage of data analysis. In 26 articles (40.6%) bracketing was used at both the data collection and the data analysis stages.

All articles surveyed implicitly employed eidetic reduction for grasping the essential meanings or features, and did so by following the stepwise method of Colaizzi [3] or Giorgi [18]. As far as grasping the essential meaning of the participants’ lived experience was concerned, 49 articles (76.6%) mentioned the identification of a common theme, 22 articles (34.4%) mentioned the identification of a hidden meaning, and 10 articles (15.6%) mentioned the use of imagination or reflection. All 64 articles employed abstraction in effecting eidetic reduction. The identification of essential meaning in the Korean phenomenological research thus was taken to be the identification of common meanings, through the abstraction of the ideas, based on the data collected.

## Discussion

Our meta-study shows diverse aims of researches. Fourteen articles (21.9%) aim at no more than a detailed description of or exploration of the experiences of participants. This shows that many researchers believe that phenomenological nursing research is merely an in-depth description of people’s lived experiences. But Husserlian phenomenology aims at grasping essential meanings by intuiting the invariant and necessary features of our experience [10]. These features are such that without them, the experience is not conceivable at all, that is, not even capable of being thought of as that experience. A mere in-depth description of people’s lived experience may be interesting, maybe important for some purposes, may shed light on a particular patient or group of patients, but is definitely not phenomenology. However, following the lead of Husserlian phenomenology, scientific phenomenology strives to grasp the essential meanings of people’s lived experience. For example, the stepwise method of Giorgi [18] aims to capture the essential description of the experience of participants, while the method of Colaizzi [3] aims to generate dense descriptions of the experiences of participants, which captures the essential aspects of those experiences. Most of the articles surveyed did indeed correctly identify the aim of phenomenological research in nursing, but some (21.9%) did not. Therefore, the articulation of the aim of phenomenological research should include the essential meaning or features of experience, and that aim, and not a general and vague aim in the near vicinity, should be a guiding principle.

Bracketing is another key concept in phenomenological methodology. Fifty-three articles (82.8%) employ researchers’ suspension of their own pre-assumptions and presuppositions respecting the subject of their research. This shows that most researchers understand the significance of bracketing and understand that bracketing is imperative for researchers themselves.

There are, however, 2 major problems involving bracketing. The first problem is incompleteness. Many researchers in the surveyed articles produce open or semi-structured interview questions (62.5%), do not interfere with participants’ narrations (32.3%), and/or try to provide a comfortable environment for the narration (59.4%). Researchers’ interviewing efforts, however, do not amount to complete researcher-centered bracketing. Complete bracketing requires an articulated and guided procedure for researchers to free themselves from all theoretical pre-assumptions and presuppositions. Such researcher-centered bracketing cannot be found in the survey articles.

A few researchers are aware of this problem and have addressed the need to develop more complete and epistemologically secure phenomenological methods of researcher-centered bracketing. For example, Tufford and Newman [19] in 2010 claim that writing a “reflexive journal” is needed throughout research as a continuous self-reflective awareness process. Ahern [20] in 1999 suggests 10 tips for “reflexive bracketing.” Their suggestions are helpful starting points for strengthening researcher-centered bracketing. Future Korean scientific phenomenological research should test them, and if effective, utilize them to ensure more successful bracketing.

The other problem is that researchers’ efforts to provide a positive atmosphere both for participants’ narration and for researchers’ bracketing do not ensure access to the pre-suppositionless and pre-assumptionless experiences. This is because participants’ narration of their experiences can be limited or tainted by their own theoretical, subjective/personal, or arbitrary pre-assumptions and presuppositions. This is the problem of the possible subjectivity of participants’ experience, a problem Crotty [6] noted as a methodological problem of scientific phenomenology in general.

Crotty [6] pointed out that scientific phenomenology is based on a misunderstanding of the participants’ subjective experiences: the data collected for analysis are the verbatim transcripts of the subjective experiences of the participants. If that is what the data amount to, subjectivity is still present, and objective and universal essences cannot be intuited by researchers’ third-person bracketing. In scientific phenomenology, the participants’ subjective experience is collected and analyzed by a third party, the researcher. The researcher brackets her own presuppositions and pre-assumptions relevant to the research subject. Such bracketing does not contribute to the elimination of subjectivity and arbitrariness in the experiences of the participants. The researcher-centered and third-person bracketing which scientific phenomenology employs thus has a fundamental gap in its methodology. It is a very significant gap, since many Korean phenomenological nursing researchers exclusively engage in researcher-centered bracketing, not fully appreciative of the critique on bracketing.

Korean phenomenological nursing researchers disagree with each other on when bracketing is appropriate. The study finds that 28.2% of the surveyed articles employ bracketing only during the data collection stage and 14.1% of the articles report its use of bracketing only during the data analysis stage. In 40.6% of the articles bracketing is used both during the data collection stage and during the data analysis stage. An article by Chan et al. [21] in 2013 argues that bracketing should be employed in initiating the research proposal, as well as both during the interview stage and during the data analysis stage. If bracketing is understood as a means to ensure the objectivity of researchers, they are correct that researchers should maintain an objective attitude during all stages of research.

Researchers’ third-person bracketing, no matter when performed and no matter how non-subjective, may well fail to result in knowledge. For without the first-person bracketing of the participants, their description of their experiences is liable to be tainted with subjectivity, prejudiced due to the intrusion of idiosyncratic or personal or theoretical presuppositions.

Eidetic reduction is an essential concept in phenomenological methodology, but the term is barely mentioned in Korean phenomenological nursing research. Colaizzi [3] and Giorgi [18] replace eidetic reduction with a stepwise analysis method which they take to result in the identification of essential meanings. Eidetic reduction is assumed to be embodied in their stepwise method [22,23].

But is it? Do the stepwise or formulaic methods of scientific phenomenology constitute eidetic reduction? The question is never asked by Colaizzi [3] or Giorgi [18], and never answered by anyone. Eidetic reduction involves the identification and removal of any and all contingent and accidental features of experiences from the first person perspective in order to intuit the invariant and necessary features of our experiences [9]. The essential features of an object in our experience are necessary and universal, without which the experience is unconceivable. Giorgi [24] in 2007 claims, without argument or explanation, that his method is a modified phenomenological method which captures the essences of human experiences. Scientific phenomenology’s assumption that the essential meanings of the experiences of participants can be grasped by the common themes and hidden meaning researchers obtain through abstraction from the fully expressed descriptions of participants’ experiences are not easily justified. The stepwise method of scientific phenomenology identifies and records themes from the descriptions of the lived experiences of participants. The danger is that the comprehensive and dense description that results may incorporate abstract and general, not necessary and universal, descriptions of the subjective experiences of participants.

Munhall [25] in 2007 has a similar concern. She points out that the stepwise methods of scientific phenomenology have led nursing research to “a form of reductionism” and to “logical positivism.” She claims that scientific phenomenology, as currently practiced, leaves researchers puzzled as to how structural methods are a part of phenomenological research, especially when, for example, data analysis results in “lists of themes, lists of essences, structural definitions, categories of abstractions, meaning units, and other reductionistic descriptions of experience.”

We are not alone in thinking that scientific phenomenology has departed from its Husserlian roots in several respects and that such departure weakens the epistemic foundation of scientific phenomenology. Crotty [6] and Paley [7] have also criticized the methodology of scientific phenomenology. Crotty [6], for example, thinks that scientific phenomenology is based on a misunderstanding of Husserl’s phenomenology. North American nursing researchers have debated the criticism of Crotty [6] and Paley [7], and have responded in various ways. Giorgi [13], himself a critic of the use of phenomenology, rejects objections of Crotty [6] and advocates the use of a more standard empirical methodology, not Husserlian phenomenology. This, however, can be read as conceding the validity of the criticism, and advocating changing the research agenda.

What exactly should researchers make of all this? Due to the vagueness of many phenomenological concepts and the lack of clear methods of application of philosophy to empirical research, phenomenological nursing researchers rarely refer to or directly utilize philosophical phenomenology in their own research. Cohen and Omery [26] in 1993 state: “Most often [they] cite secondary sources to reference their methods.” All 64 articles we surveyed follow either the data analysis method of Giorgi [18] or of Colaizzi [3]; and all follow the global trend of referring to Giorgi [18] or of Colaizzi [3] as the primary source of their phenomenological methodology. There can be no doubt that their methods have produced results. As Munhall [25] in 2007 points out, they have brought “phenomenology into the academy with rules.” Phenomenological nursing research, as qualitative research, has gained its popularity not because of pure Husserlian phenomenology, but thanks to the methods of scientific phenomenology of Giorgi [18] and Colaizzi [3]. Thus it is hard to know what to make of the fact that, as Cohen and Omery [26] in 1993 point out, it has not been shown, or even made open to critical discussion, whether the data analysis of scientific phenomenology results in the sorts of secure knowledge outcomes which scientific phenomenology aims at producing by grounding itself on Husserlian phenomenology.

### Implications for descriptive phenomenological nursing research

We believe that the philosophical rigor of the Husserlian phenomenology can be incorporated into scientific phenomenology, and that the result will be more secure scientific knowledge. Rather than choosing either philosophical phenomenology or scientific phenomenology, as Barkway [27] in 2001 suggests, we propose an adaptation of the methodology of Crotty [6]. This adaptation emphasizes participants-centered bracketing and participant-centered eidetic reduction in addition to rigorous researcher-centered bracketing and eidetic reduction and integrates them into scientific phenomenology. The major objection of Crotty [6] and Paley [7] concerns the subjectivity of the outcome of the scientific phenomenological research. But an elementary point needs to be fully appreciated: In the empirical sciences, such as nursing, it may be impossible to establish universal and objective knowledge. Cartesian certainty may not be achievable. Nursing is not mathematics. On the other hand, equally importantly, scientific phenomenology can assure universal and objective knowledge when researchers work on data collected from the subjectivity-cleansed and reflected experiences of participants gathered from their first-person perspectives.

We think that the elimination of much subjectivity at the stages of data collection and data analysis is possible but that doing so requires helping participants perform first-person bracketing and first-person eidetic reduction. Researchers’ own bracketing in the data collection stage and particularly in data analysis stage is required. In addition, to discover the essential meanings of the experience of participants, each participant must, from a first-person perspective, bracket her unexamined and taken-for-granted assumptions and presuppositions. Furthermore, each participant must also perform an eidetic reduction from that perspective to help researchers intuit the essential features of her experiences.

Participant-centered bracketing and eidetic reduction can be integrated into interviewing questions and interviewing procedures. Some nursing researchers have already incorporated participants-centered bracketing and eidetic reduction into their data collection procedures. For example, the method of Seidman [28] in 2006 of interviewing requires 3 interviews for each participant, one of which focuses on the respondent’s reflection on the meaning of his or her experience. This interview stage includes the first-person eidetic reduction. Bevan [15] presents an outline of phenomenological interviewing. In the outline, he includes first-person eidetic reduction in such questions as: “Describe how the unit experience would change if a doctor was present at all times?” Bevan acknowledges the difficulty of implementing “imaginative variation” in first-person eidetic reduction in the context of an interview. This difficulty also attends the implementation of first-person bracketing in the context of an interview. The solution of Bevan [15] is to generate variational questions in order to generate first-person eidetic reduction during multiple interviews. The proposals by Seidman [28] in 2006 and Bevan [15] in 2014 are promising. They indicate directions for developing participant-centered bracketing and eidetic reduction, though, as always, testing for effectiveness is needed if they are to prove their worth. We also believe that researchers can guide the participants to bracket their own presuppositions and assumptions during multiple interviews. What is absolutely required is researchers’ own bracketing. The rigorous practice of researchers’ own bracketing will enable them to formulate interview questions and guide interviews for participants’ bracketing. In this way, researchers can identify the essential features of the experiences of participants and eliminate more subjective, contingent, and arbitrary data.

### Conclusion

Korean phenomenological nursing research exemplified the enthusiasm for and the potential of scientific phenomenology in nursing research. In Korea, researchers have recognized the promise of scientific phenomenology and the need for further development which is solidly philosophically founded. Both the quantity and the quality of scientific phenomenological research in Korea showed its promise on the global stage. But despite its promise, descriptive phenomenological methodology has remained simply the brand of Colaizzi [3] and Giorgi [18] of scientific phenomenology. This is true not just in Korea but across the globe. There has been almost no methodological development, especially in relation to Husserl’s philosophical principles. Korean researchers must revisit the methodology of scientific phenomenology and discuss ways to increase objectivity. This paper suggests that by incorporating Husserlian participant-centered bracketing and participant-centered-person eidetic reduction into scientific phenomenological nursing research to a much greater extent than at present, Korean nursing scholars can contribute to the accumulation of nursing knowledge with a philosophically secure and rigorous foundation.

## Figures and Tables

**Table 1.** Framework for critical review of phenomenological nursing research

Criteria	Category	Features
General characteristics		- Published year
		- Type of participants
		- Method of sampling
Descriptive qualitative research features	Interview type	- In-depth individual interview
		- Combined with focused interview
		- Combined with structured questionaire
	Question type	- Open
		- Semi-structured
		- Structured
	Enhancing quality of interview data	- Comfortable environment provided to the participants
		- Rapport between researcher and participants established
		- Non-interference with participants’ narration
	Validity criterion	- Lincoln and Guba/Sandelowski/other criteia
	Truthfulness	- Member check/peer check
Phenomenological research features	Purpose/aim	- Rich description of experience
		- Essential meaning and structure of experience
	Phenomenological reduction (bracketing)	- Researcher’s suspension of their own pre-assumptions and presuppositions
	Method of data analysis	- Giori/Colaizzi’s phenomenological method
	Eidetic reduction	- Identification of a common theme
		- Use of imagination or reflection
		- Identification of a hidden meaning
		- Abstraction

**Table 2.** Frequencies of the researches by the characteristic of the study

Criteria	Features	No. (%)
General characteristics		
Published year	2005	4 (4.7)
	2006	3 (4.7)
	2007	4 (6.3)
	2008	4 (6.3)
	2009	4 (6.3)
	2010	4 (6.3)
	2011	5 (7.8)
	2012	4 (6.3)
	2013	5 (7.8)
	2014	9 (14.1)
	2015	5 (7.8)
	2016	3 (4.7)
	2017	11 (17.2)
	2018	3 (4.7)
Type of participants	Patients	24 (37.5)
	Nurses	18 (28.1)
	Nursing students	7 (10.9)
	Family	4 (6.3)
	Elderly	7 (10.9)
	Others (clinical workers, homeless, & immigrants)	4 (6.5)
Method of sampling	Convenient	11 (17.2)
	Purposive	16 (25.0)
	Convenient or purpose with snowballing	12 (18.8)
	No mention	25 (39.1)
Descriptive qualitative research features		
Interview type^a)^	In-depth individual interview	64 (100.0)
	Combined with focused interview	7 (10.9)
	Combined with structured questionnaire	3 (4.7)
Question type	Open	13 (20.3)
	Semi-structured	9 (14.1)
	Structured	1 (1.6)
	Open+semi-structured	18 (28.1)
	Unknown	23 (35.9)
Enhancing quality of interview data^a)^	Comfortable environment	38 (59.4)
	Established rapport	37 (58.8)
	Non-interference with participants’ narration	20 (32.3)
Validity criterion	Lincoln and Guba’s criteria	33 (51.6)
	Sandelowski’s criteria	13 (20.3)
	Other	4 (6.3)
	No comment	14 (21.9)
Truthfulness^a)^	Member check (a)	39 (60.9)
	Peer check (b)	24 (37.5)
	(a) and (b)	15 (23.4)
	(a) or (b)	48 (75.0)
Phenomenological research features		
Purpose/aim	Rich description	14 (21.9)
	Essential meaning or structure	50 (78.1)
Occurrence of bracketing^a),b)^	During data collection (c)	44 (68.8)
	During data analysis (d)	35 (54.7)
	(c) and (d)	26 (40.6)
	(c) or (d)	53 (82.8)
Method of data analysis	Giorgi’s phenomenological method	17 (26.6)
	Colaizzi’s phenomenological method	47 (73.4)
Eidetic reduction^a)^	Identification of a common theme	49 (76.6)
	Use of imagination or reflection	10 (15.6)
	Identification of a hidden meaning	22 (34.4)
	Abstraction	64 (100.0)

a) Multiple responses.

b) Bracketing: researchers’ suspension of their own pre-assumptions and presuppositions; a (member check); b (peer check); c (during data collection); and d (during data analysis).

## Supplementary materials

Supplement 1. The audio recording of the abstract.
